# Supplementary figures and images for: A spatial predictive model for malaria resurgence in central Greece integrating entomological, environmental and social data
Source: PLoS One. 2017 Jun 29;12(6):e0178836. doi: 10.1371/journal.pone.0178836 (PMC5490999; doi:10.1371/journal.pone.0178836)

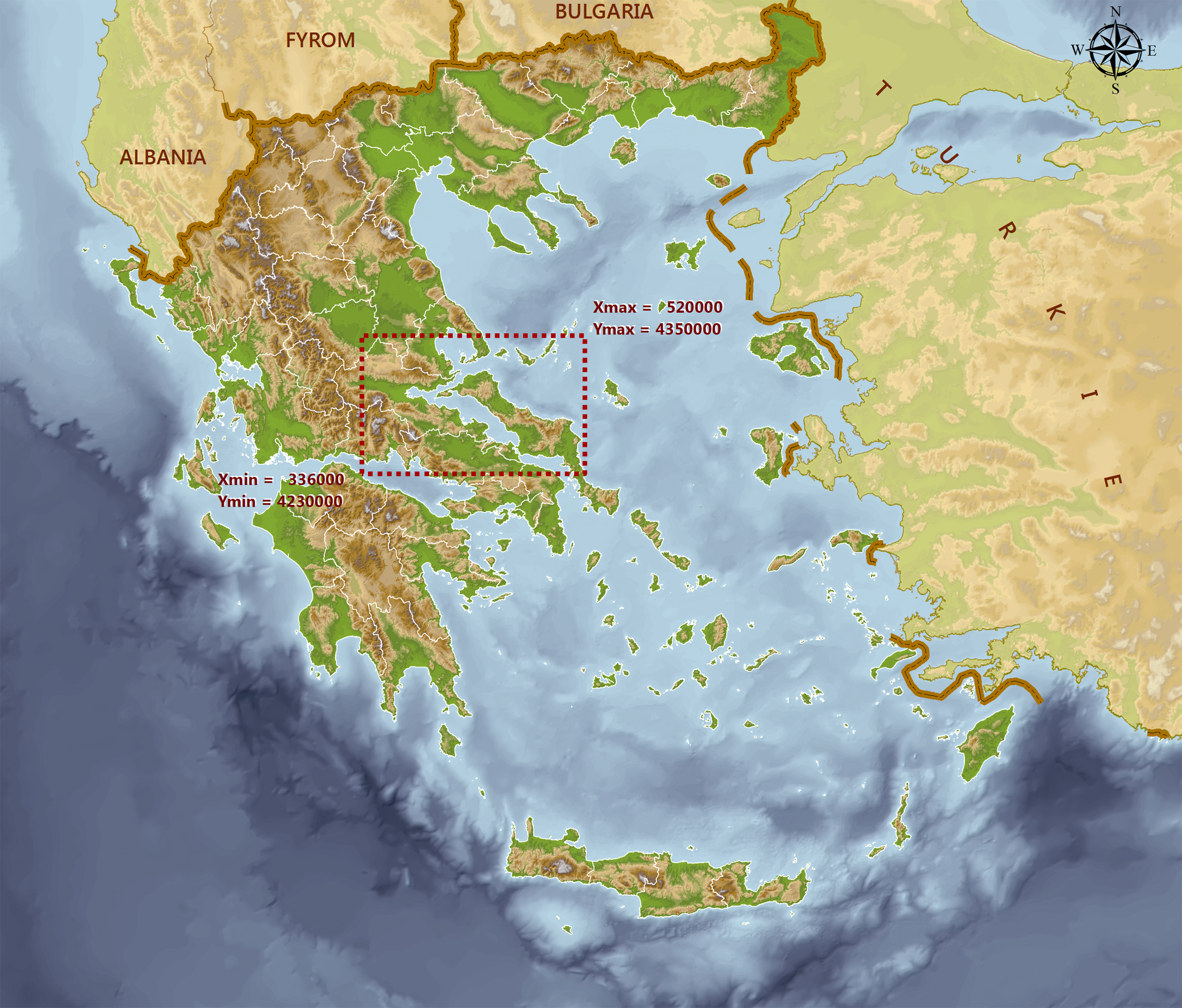

Supplement: S1 Fig — (TIF) [file pone.0178836.s001.tif]

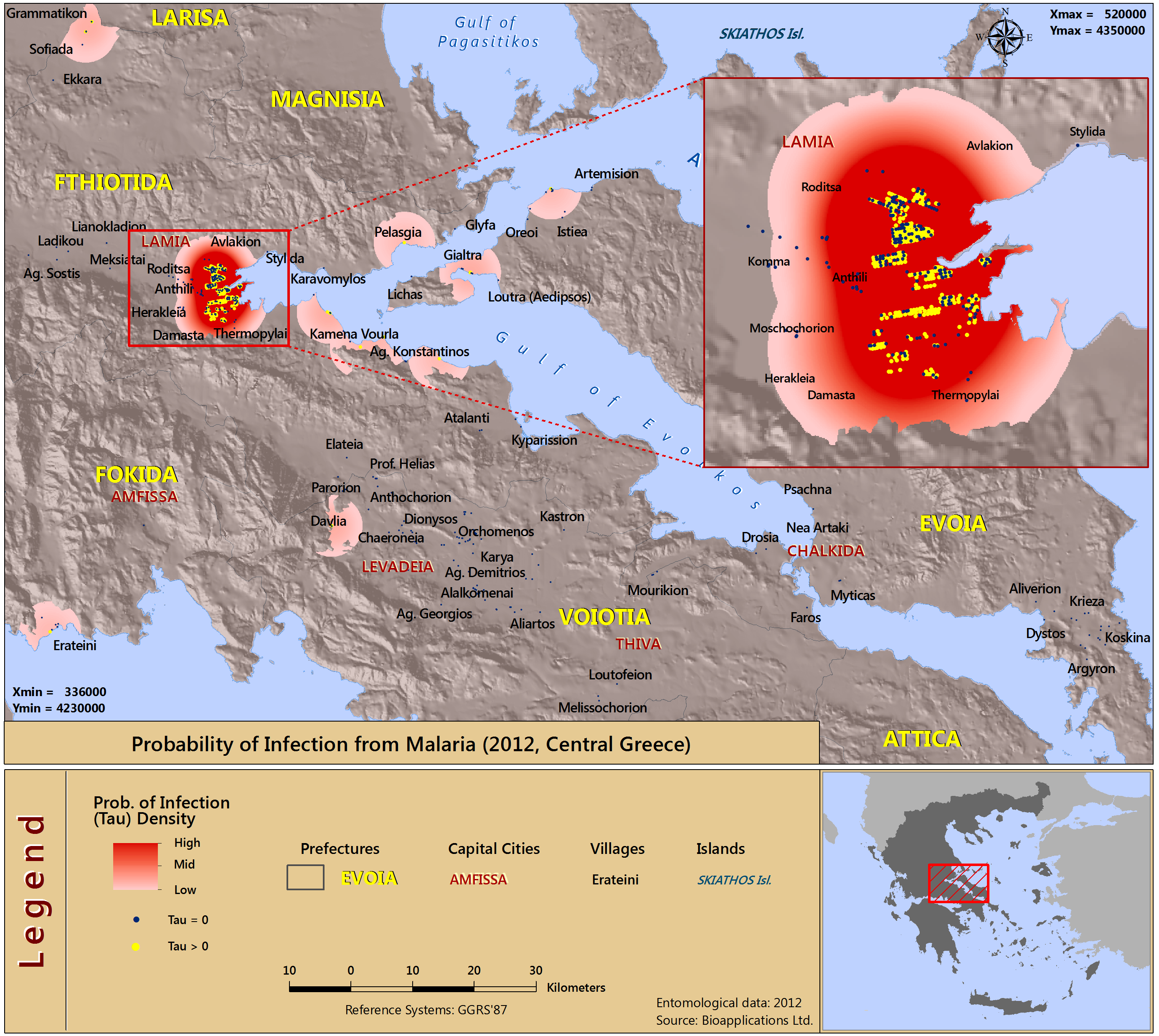

Supplement: S2 Fig — (TIF) [file pone.0178836.s002.tif]

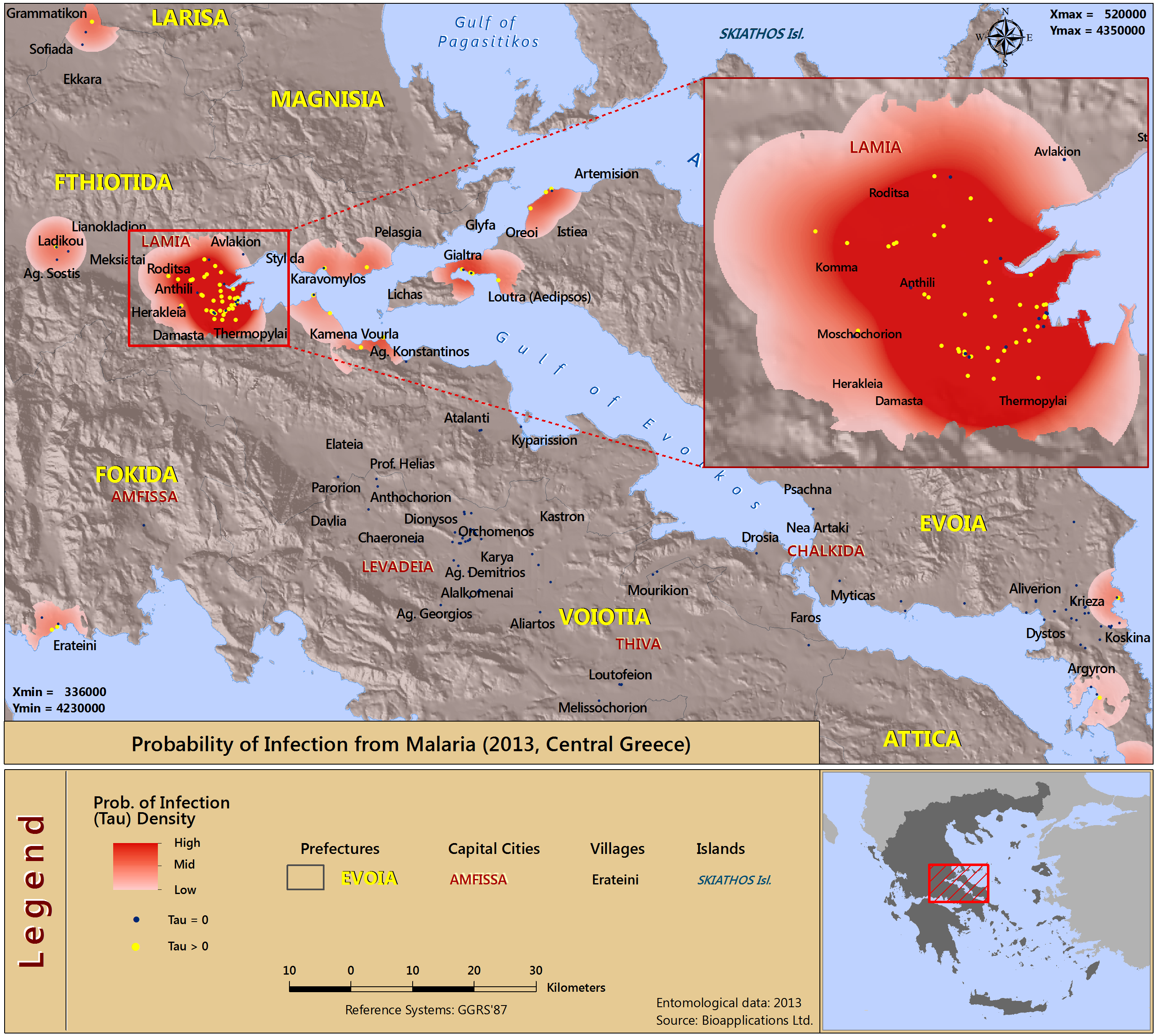

Supplement: S3 Fig — (TIF) [file pone.0178836.s003.tif]

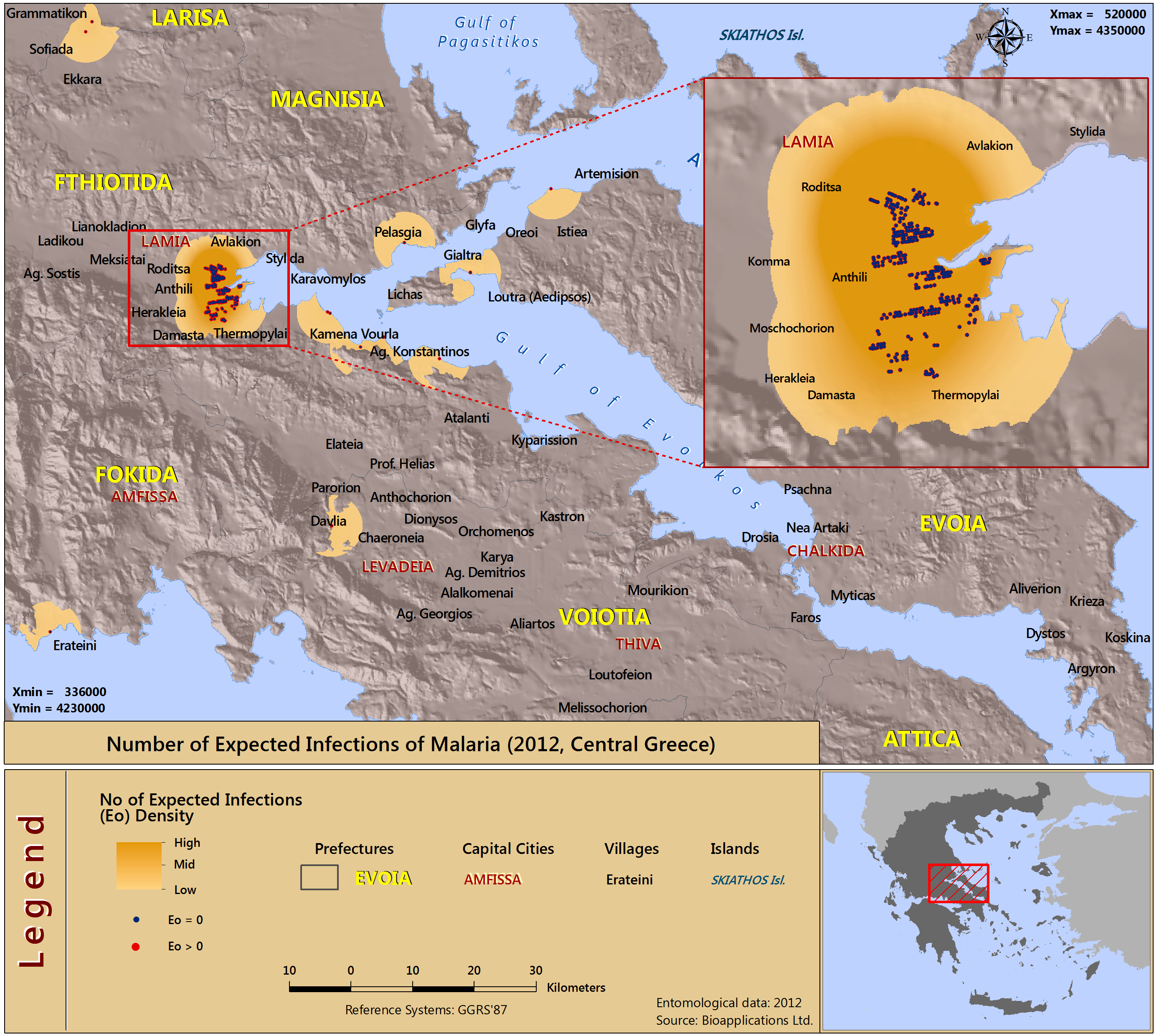

Supplement: S4 Fig — (TIF) [file pone.0178836.s004.tif]

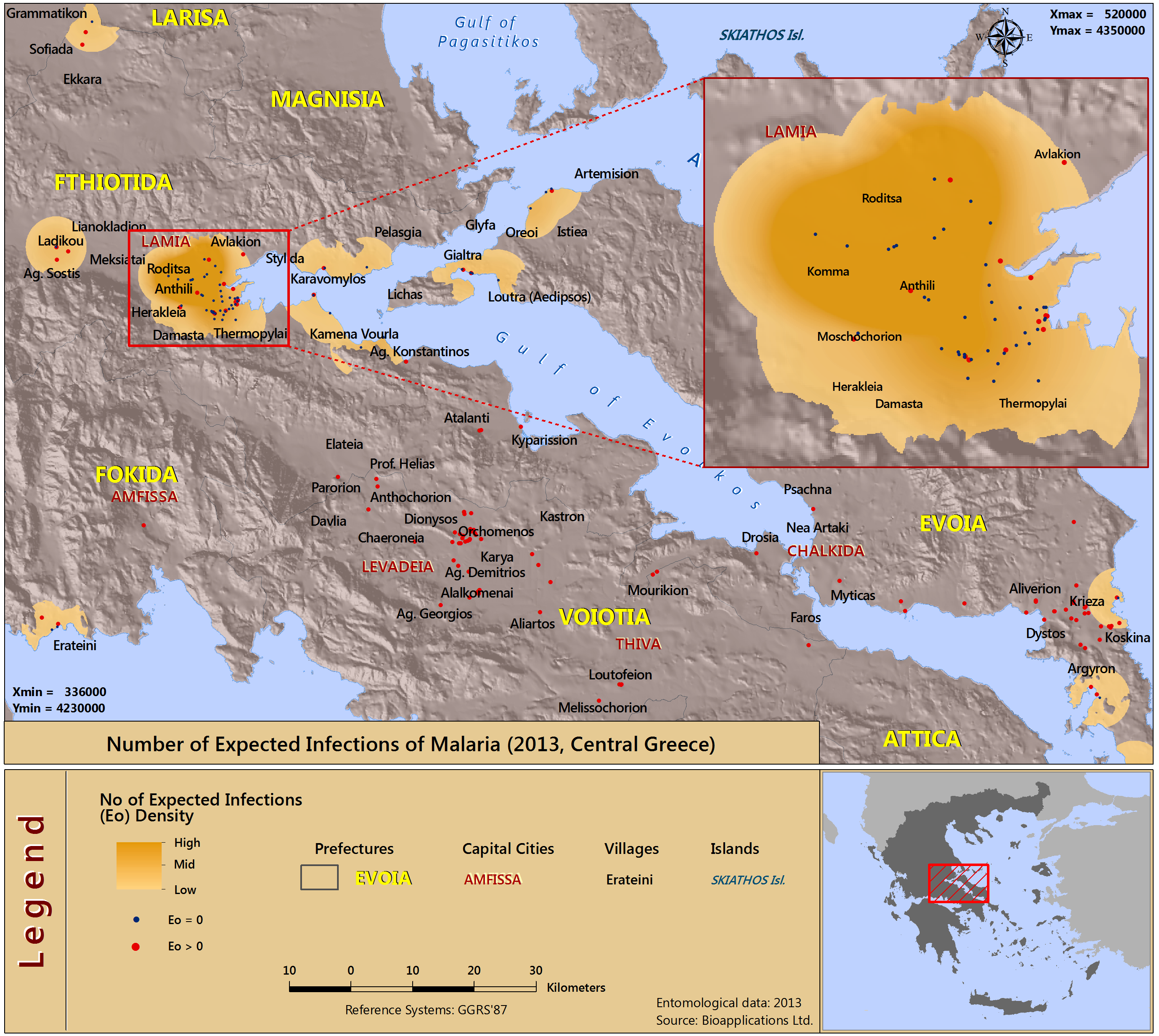

Supplement: S5 Fig — (TIF) [file pone.0178836.s005.tif]
