# Supplementary material for: A spatial predictive model for malaria resurgence in central Greece integrating entomological, environmental and social data
Source: PLoS One. 2017 Jun 29;12(6):e0178836. doi: 10.1371/journal.pone.0178836 (PMC5490999; doi:10.1371/journal.pone.0178836)
Supplement: S1 Table — (DOCX) [file pone.0178836.s006.docx]

| **Parameter or variable** | **Interpretation** | **Distribution/value** |
| --- | --- | --- |
| $\alpha_{i}$ | % of mosquitoes that feed on humans/day | Uniform(0.01, 0.5) |
| $b_{i}$ | Probability a bite produces infection to a human | Uniform(0.2, 0.5) |
| c | Probability a bite turns a susceptible mosquito to infected | 0.5 |
| $r_{i}$ | Average daily recovery rate/day | Uniform(0.01, 0.5) |
| $v_{i}$ | # of days from infection to infectiousness in the mosquito | Uniform(5, 15) |
| $m_{i}$ | The ratio of mosquitoes to humans in region *i* | Observed data |
| $g_{i}$ | Mosquito mortality rate/day | $g_{i}=f\left(T_{i}\right)$ |
| $T_{i}$ | Average temperatures | Observed data |
| $alpha$ | Spatial parameter for modeling the spatial component of migrant transmission | Observed data |
| $d_{ik}$ | Distances of migrant population from the larvae areas | Observed data |

**S1 Table.** List of model parameters and interpretations
