# Supplementary material for: A spatial predictive model for malaria resurgence in central Greece integrating entomological, environmental and social data
Source: PLoS One. 2017 Jun 29;12(6):e0178836. doi: 10.1371/journal.pone.0178836 (PMC5490999; doi:10.1371/journal.pone.0178836)
Supplement: S2 Table — (DOCX) [file pone.0178836.s007.docx]

|  |  | **Apr** | **May** | **Jun** | **Jul** | **Aug** | **Sep** | **Oct** | **Nov** |
| --- | --- | --- | --- | --- | --- | --- | --- | --- | --- |
| **2012** | **Total** | 0.000 | 0.087 | 0.099 | 0.100 | 0.100 | 0.100 | 0.100 | 0.000 |
|  | **Urban** | 0.000 | 0.000 | 0.000 | 0.000 | 0.000 | 0.000 | 0.000 | 0.000 |
|  | **Rural** | 0.038 | 0.095 | 0.100 | 0.100 | 0.100 | 0.100 | 0.100 | 0.000 |
| **2013** | **Total** | 0.032 | 0.033 | 0.036 | 0.038 | 0.038 | 0.038 | 0.024 | -- |
|  | **Urban** | 0.000 | 0.000 | 0.000 | 0.000 | 0.000 | 0.000 | 0.000 | -- |
|  | **Rural** | 0.035 | 0.036 | 0.039 | 0.041 | 0.040 | 0.040 | 0.028 | -- |

**S2 Table.** Median $\tau$ by month/region type (Years 2012 & 2013).
