# Supplementary material for: A spatial predictive model for malaria resurgence in central Greece integrating entomological, environmental and social data
Source: PLoS One. 2017 Jun 29;12(6):e0178836. doi: 10.1371/journal.pone.0178836 (PMC5490999; doi:10.1371/journal.pone.0178836)
Supplement: S3 Table — (DOCX) [file pone.0178836.s008.docx]

|  |  | **Apr** | **May** | **Jun** | **Jul** | **Aug** | **Sep** | **Oct** | **Nov** |
| --- | --- | --- | --- | --- | --- | --- | --- | --- | --- |
| **2012** | **Total** | 0.0 | 15.0 | 17.1 | 17.2 | 17.2 | 17.2 | 17.2 | 0.0 |
|  | **Urban** | 0.0 | 0.0 | 0.0 | 0.0 | 0.0 | 0.0 | 0.0 | 0.0 |
|  | **Rural** | 6.5 | 16.3 | 17.2 | 17.2 | 17.3 | 17.3 | 17.2 | 0.0 |
| **2013** | **Total** | 20.5 | 21.1 | 23.3 | 24.3 | 24.1 | 24.2 | 15.5 | -- |
|  | **Urban** | 0.0 | 0.0 | 0.0 | 0.0 | 0.0 | 0.0 | 0.0 | -- |
|  | **Rural** | 22.6 | 23.0 | 25.0 | 26.1 | 25.9 | 26.0 | 18.1 | -- |

**S3 Table.** Median E(infection) by month/region type (Years 2012 & 2013).
