# Supplementary material for: A spatial predictive model for malaria resurgence in central Greece integrating entomological, environmental and social data
Source: PLoS One. 2017 Jun 29;12(6):e0178836. doi: 10.1371/journal.pone.0178836 (PMC5490999; doi:10.1371/journal.pone.0178836)
Supplement: S4 Table — (DOCX) [file pone.0178836.s009.docx]

| **sensitivity** | **2012** | | | **2013** | | |
| --- | --- | --- | --- | --- | --- | --- |
|  | $R_{0}$ | $\tau$ | E(infection) | $R_{0}$ | $\tau$ | E(infection) |
| **5%** | 0.236 | 0.026 | 7.3 | 1.037 | 0.022 | 20.98 |
| **10%** | 0.408 | 0.037 | 12.46 | 1.074 | 0.025 | 21.8 |
| **20%** | 0.755 | 0.058 | 23.18 | 1.144 | 0.028 | 23.26 |

**S4 Table.** Median estimates of$R_{0}$, $\tau$ and E(infection) for the various scenario specifications of the baseline prevalence.
