# Supplementary material for: A spatial predictive model for malaria resurgence in central Greece integrating entomological, environmental and social data
Source: PLoS One. 2017 Jun 29;12(6):e0178836. doi: 10.1371/journal.pone.0178836 (PMC5490999; doi:10.1371/journal.pone.0178836)
Supplement: S3 File — (DOCX) [file pone.0178836.s012.docx]

The method used to represent the phenomenon was “Kernel Density” (Spatial Analyst of ArcGIS – ArcMap v.10.x). The Density tool distributes a measured quantity of an input point layer throughout a landscape to produce a continuous surface. Density surfaces show where point features are concentrated. By calculating density, you are in a sense spreading the values (of the input) out over a surface. The magnitude at each sample location is distributed throughout the study area, and a density value is calculated for each cell in the output surface.

The Kernel Density tool calculates the density of features in a neighborhood around those features. The population field (in our case the values of median Ro, Expected # of infections and τ) could be used to weight some features more heavily than others, depending on their meaning, or to allow one point to represent several observations. The resulting surfaces surrounding each point in kernel density are based on a quadratic formula with the highest value at the center of the surface (the point location) and tapering to zero at the search radius distance. For each output cell, the total number of the accumulated intersections of the individual spread surfaces is calculated. The way of density calculation depends on a bandwidth that uses a default search radius algorithm. This algorithm:

1. Calculates the mean center of the input points. If a Population field other than “None” was selected, this, and all the following calculations, will be weighted by the values in that field.
2. Calculates the distance from the (weighted) mean center for all points.
3. Calculates the (weighted) median of these distances, D_m_.
4. Calculates the (weighted) Standard Distance, SD (see below).
5. Applies the following formula to calculate the bandwidth:


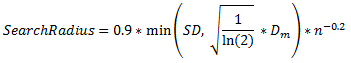


where:

- - SD is the standard distance
  - D_m_ is the median distance
  - n is the number of points if no population field is used, or if a population field is supplied, n is the sum of the population field values

Note that the min part of the equation means that whichever of the two options that results in a smaller value will be used. This approach to calculating a default radius generally avoids the "ring around the points" phenomenon that often occurred with sparse datasets.

Standard Distance measures the compactness of a distribution provides a single value representing the dispersion of features around the center. The value is a distance, so the compactness of a set of features can be represented on a map by drawing a circle with the radius equal to the standard distance value. The standard distance is given as:


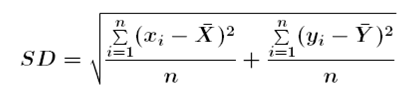


where:

- x_i_ and y_i_ are the coordinates of feature (point) i,
- $\overline{X}$ and $\overline{Y}$ represents the mean center for the features (points)
- n is equal to the total number of features

In our case *SearchRadius* was calculated about 5,000 meters due to our research area (Prefectures of Fokida, Voiotia, Fthiotida and Evoia). In other words *SearchRadius* affected by the research area and its size shrinks or grows proportionately according to the size of this area. An altitude zone which represents the areas being between 0 and 300 m was situated as a threshold in order to avoid the depiction of the density in areas which are not affected from the represented phainomenon.

The output values represent the predicted density value per square kilometers.

References:

Silverman, B. W., 1986. Density Estimation for Statistics and Data Analysis. New York: Chapman and Hall.

ESRI™, 1995-2014. ArcGIS v. 10.3 User Manual, ESRI Redlands.
